# Vector-independent transmembrane transport of oligodeoxyribonucleotides involves p38 mitogen activated protein kinase phosphorylation

**DOI:** 10.1038/s41598-017-14099-0

**Published:** 2017-10-19

**Authors:** Minyuan Peng, Yanming Li, Jian Zhang, Yong Wu, Xiaoyang Yang, Ye Lei, Mao Ye, Jing Liu, Xu Han, Yijin Kuang, Xielan Zhao, Fangping Chen

**Affiliations:** 1 Hematology Department, Xiangya Hospital, Central South University, Changsha, 410008 Hunan China; 2 Laboratory Department, Xiangya Hospital, Central South University, Changsha, 410008 Hunan China; 3 Hematology Department, The Third Xiangya Hospital, Central South University, Changsha, 410013 Hunan China; 4 Urology surgery Department, Xiangya Hospital, Central South University, Changsha, 410008 Hunan China; 5 Molecular Science and Biomedicine Laboratory, State Key Laboratory for Chemo/Biosensing and Chemometrics, College of Biology, College of Chemistry and Chemical Engineering, Hunan University, Changsha, 410082 Hunan China; 6 Molecular Biology Research Center, School of Life Science, Central South University, Changsha, 410078 China

## Abstract

The main roles of equilibrative nucleoside transporters (ENTs) and concentrative nucleoside transporters (CNTs) are to transfer single nucleosides and analogues for the nucleic acid salvage pathway. Oligodeoxyribonucleotides (ODNs) can be transported into the cytoplasm or nucleus of cells under certain conditions. Among ODNs composed of a single type of nucleotide, the transport efficiency differs with the length and nucleotide composition of the ODNs and varies in different types of leukaemia cells; among the 5 tested random sequence ODNs and 3 aptamers with varying sequences, the data showed that some sequences were associated with significantly higher transport efficiency than others. The transport of ODNs was sodium, energy, and pH-independent, membrane protein-dependent, substrate nonspecific for ODNs and 4-nitrobenzylthioinosine (NBMPR)-insensitive, but it showed a low sensitivity to dipyridamole (IC50 = 35.44 µmol/L), distinguishing it from ENT1-4 and CNTs. The delivery efficiency of ODNs was superior to that of Lipofection and Nucleofection, demonstrating its potential applications in research or therapeutics. Moreover, this process was associated with p38 mitogen activated protein kinase (p38MAPK) instead of c-Jun N-terminal kinase (JNK) signalling pathways. We have denoted ODN transmembrane transport as equilibrative nucleic acid transport (ENAT). Overall, these findings indicate a new approach and mechanism for transmembrane transport of ODNs.

## Introduction

Despite the remarkable progress in the field of gene therapy for haematological diseases during the past several decades, limitations in gene delivery methods represent a barrier to its clinical application. Major limitations lie in low gene delivery efficiency due to the macromolecular and poly-anionic properties of the nucleic acids and the questionable safety of the delivery vectors^1^. Among various virus- or non-virus-based carrier systems, well-designed viral vectors (*e.g*., lentiviral vectors or adeno-associated virus [AAV] vectors) represent the gene delivery tools with the highest efficiency^2^. However, most of these vectors are associated with significant disadvantages. Lentiviral vectors can effectively integrate into the host cell genome, but genome integration may cause leukaemia if the insertion site is adjacent to a proto-oncogene^3,4^. AAV vectors can be applied to a broad range of hosts with very few insertion mutations, but they may induce a capsid-specific T cell immune response^5,6^ and integration-mediated tumourigenicity^7^. Non-virus (*e.g*., chemical vector) methods have shown a high delivery efficiency of Oligodeoxyribonucleotides (ODN) transport^8–10^, but there is a lack of long-term safety data^1,11^. Moreover, some physical methods may require expensive apparatuses or cause damage to samples^11^. Therefore, effective and safe gene delivery remains an area of improvement in gene therapy.

ODNs, short single-stranded DNA (ssDNA) molecules, have a broad range of applications in the field of targeted therapy. Single-stranded antisense ODNs can form DNA/RNA hybrids with specific mRNA strands to mediate mRNA degradation and can therefore be used to silence target gene expression^12^. Nucleic acid aptamers, oligonucleotide molecules that bind to a specific target molecule (protein or RNA) with high affinity, can be used to identify tumour cell biomarkers or mediate gene silencing for therapeutic purposes^13–17^. However, the delivery of ODNs into cells with high efficiency, especially without the aid of vectors, is very challenging.

**Figure 1:**
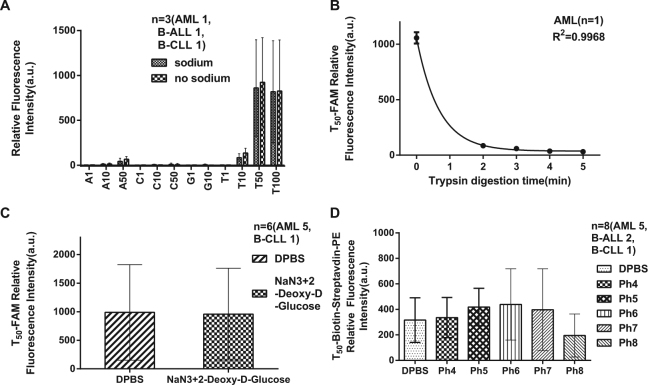
Characteristics of the transmembrane transport of ODNs. Treated clinical cells were incubated in various buffers to investigate the features of ODN transport. (**A**) In sodium or sodium-free buffer, the transmembrane transport of ODNs with different lengths and a single type of nucleotides were not significantly different (*p* > 0.05). (**B**) After trypsin digestion, the fluorescence intensity of T_50_-FAM clearly declined (*p* < 0.05), which indicated that ODN transport was membrane protein dependent. (**C**) To exhaust cellular ATP, cells were incubated in 10 mM sodium azide and 6 mM 2-deoxy-D-glucose DPBS buffer for 1 hr *vs* DPBS, and no significant difference in T_50_-FAM transport was observed in the groups (*p* > 0.05). (**D**) In gradient pH (4–8) phosphate buffer, there were no differences in T_50_-FAM transport (*p* > 0.05). All experiments were performed in triplicate or duplicate.

Previous research has reported that nucleic acids, including ODNs, can be actively internalized into living cells^18–21^. The possible mechanism is receptor- mediated, temperature-dependent endocytosis. Following entry into the endocytic pathway, the majority of ODNs are degraded by lysosomal enzymes, and only a small fraction is released into the cytoplasm^1^. Thus, endocytosis is not suitable for ODN delivery. In this study, we observed that ODNs could be transported intact into the cytoplasm without any delivery tools in a temperature and energy-independent manner (Fig. 1C), although the exact mechanism was unclear. We hypothesized that the nucleoside transporter family, which is important for cellular nucleoside uptake, was involved in ODN transport. However, we found that ODN transport differed from concentrative nucleoside transporters (CNTs) or equilibrative nucleoside transporters (ENTs), and it involved the intracellular signalling pathway p38MAPK. Here, we describe the novel transport of ODNs in cells. To our knowledge, no other studies have examined ODN transmembrane transport and the underlying mechanism.

## Results

### Characteristics of ODN transmembrane transport

Because CNTs are sodium-dependent, we assessed ODN transmembrane transport in the presence or absence of sodium. Clinical leukaemia cells (AML, B-ALL, and B-CLL) were incubated with FAM-labelled A_1_, T_1_, C_1_, G_1_, A_10_, T_10_, C_10_, G_10_, A_50_, C_50_, T_50_ and T_100_ in either sodium or sodium-free buffer^22^. Figure 1A shows that the fluorescence intensity of the cells was not significantly different between sodium and sodium-free buffer (*p* > 0.05), indicating that ODN transmembrane transport was sodium-independent. As shown in Fig. 1B, pretreatment with trypsin for 2 minutes significantly reduced the fluorescence intensity of T_50_-FAM-treated cells, which suggested that ODN transmembrane transport was membrane protein-dependent. The fluorescence intensity did not decrease further with trypsin pretreatment longer than 2 minutes, possibly because of complete destruction of membrane proteins at this time point. As shown in Fig. 1C, there were no significant differences between cells pre-treated with sodium azide and 2-deoxy-D-glucose and the control group (*p* > 0.05). This result indicated that ODN transport was a passive and energy-independent mechanism. As shown in Fig. 1D, the T_50_-biotin-streptavidin-PE fluorescence of cells in different pH buffers was not significantly different, which suggested that ODN transport was insensitive to pH.

### Transmembrane transport of ODNs in clinical leukaemia cells

**Figure 2:**
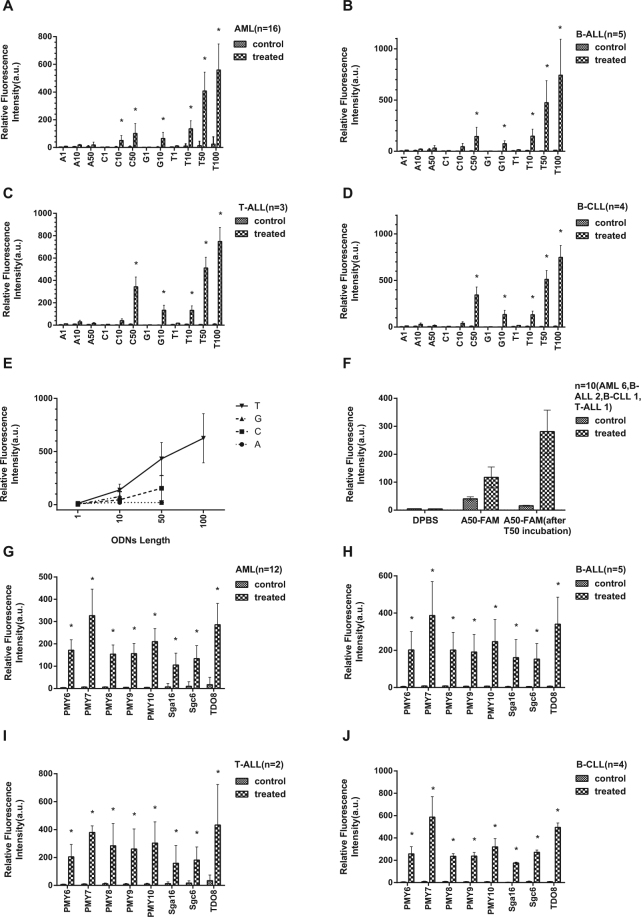
Flow cytometry quantification of the transmembrane transport efficiency of different ODNs. Clinical leukaemia cells were incubated with ODNs or DPBS for 30 minutes. Treated cells were incubated with solution A, and untreated cells were incubated with DPBS. (**A**–**D**) The fluorescence intensity of different types of clinical leukaemia cells, AML, B-ALL, B-CLL and T-ALL, incubated with various FAM-labelled ODNs composed of a single type of nucleotide *vs* control (incubated with DPBS). The transport efficiency of various ODNs, indicated by the intracellular fluorescence intensity, differed depending on the sequence and the cell type. (**E**) In the treated group, the fluorescence intensity of cells treated with FAM-labelled ODNs composed of a single type of nucleotides *vs* the length of the ODNs. The fluorescence intensity generally increased with increasing length of ODNs composed of the same type of nucleotide. (**F**) The fluorescence intensity of clinical leukaemia cells treated with FAM-labelled A_50_ with and without T_50_ pre-incubation *vs* control (incubated with DPBS). The cellular fluorescence intensity was significantly increased following T_50_ pre-incubation, indicating a higher transport efficiency of A_50_ in response to this procedure. (**G**–**J**) The fluorescence intensity of clinical AML, B-ALL, B-CLL, and T-ALL cells treated with various FAM-labelled random sequence ssDNAs (PMY_6-10_) and aptamers (sgc6, sga16, TD08) *vs* control (incubated with DPBS). While different types of cells showed varying fluorescence intensities when treated with the same ODNs, PMY_7_-FAM and TD08-FAM consistently displayed the highest fluorescence intensity among all random sequence ssDNAs and aptamers, respectively, in all cell types.

Clinical leukaemia AML, B-ALL, B-CLL and T-ALL cells can transport various FAM-labelled ODNs into the cytoplasm. Figure 2A–D indicates that ODNs composed of a single type of nucleotides were transported in all 4 types of clinical leukaemia cells without delivery vectors, and the transport efficiency varied depending on the cell type and ODN sequence. Within each cell type, the order of decreasing ODN transport efficiency was as follows: for AML: T_100_ > T_50_ > T_10_ > C_50_ > G_10_ > C_10_ > A_10_ > A_50;_ B-ALL: T_100_ > T_50_ > T_10_ > C_50_ > G_10_ > C_10_ > A_50_ > A_10;_ B-CLL: T_100_ > T_50_ > C_50_ > G_10_ > T_10_ > C_10_ > A_10_ > A_50;_ T-ALL: T_100_ > T_50_ > T_10_ > C_50_ > G_10_ > C_10_ > A_10_ > A_50_. When comparing transport efficiency across cell types, T-ALL cells generally had a lower fluorescence intensity than the other cell types for most ODNs. Figure 2E shows that the length of the ODNs affected the transport efficiency in clinical leukaemia cells (all 4 cell types mixed together). In general, the transport efficiency, as indicated by the fluorescence intensity, increased with increasing ODN length in the treated cells. Among the 10-nucleotide ODNs, T_10_ and G_10_ were transported with higher efficiency than A_10_ and C_10_ (*p* < 0.001). Among ODNs of 50–100 nucleotides, T_50_ and T_100_ demonstrated a higher transport efficiency than C_50_ and A_50_ (*p* < 0.001). Figure 2F shows that, in clinical leukaemia cells (all 4 cell types mixed together), the transport efficiency of A_50_ was increased after pre-incubation with T_50_ compared with the control. This finding seemed reasonable because T_50_ could bind and maintain a low concentration of A_50_ in the cytoplasm, thus maintaining the inward-directed A_50_ concentration gradient, which resulted in an influx of extracellular A_50_.

ODN transmembrane transport also occurred for random sequences of ssDNAs and aptamers. Figure 2G–J shows the fluorescence intensity in clinical AML, B-ALL, B-CLL and T-ALL cells treated with different random sequence ssDNAs (PMY_6-10_) and aptamers (sgc6, sga16, TD08). We found that B-CLL cells had a higher fluorescence intensity than other cell types incubated with PMY7 and TD08 (*p* < 0.05), but not other ODNs. Moreover, T-ALL cells showed the lowest fluorescence intensity among all cell types for most random sequence ssDNAs and aptamers. While the different types of leukaemia cells varied in fluorescence intensity when treated with the same ODNs, treatment with some ODNs consistently resulted in a higher fluorescence intensity than others across cell types. Among all random sequence ssDNAs, PMY_7_ treatment resulted in the highest fluorescence intensity. For aptamers, TD08 treatment was associated with higher fluorescence intensity than sgc6 and sga16. These results suggested that the transport efficiency for random sequence ssDNAs and aptamers was influenced by the DNA sequence.

### Kinetics of ODN transmembrane transport

**Figure 3:**
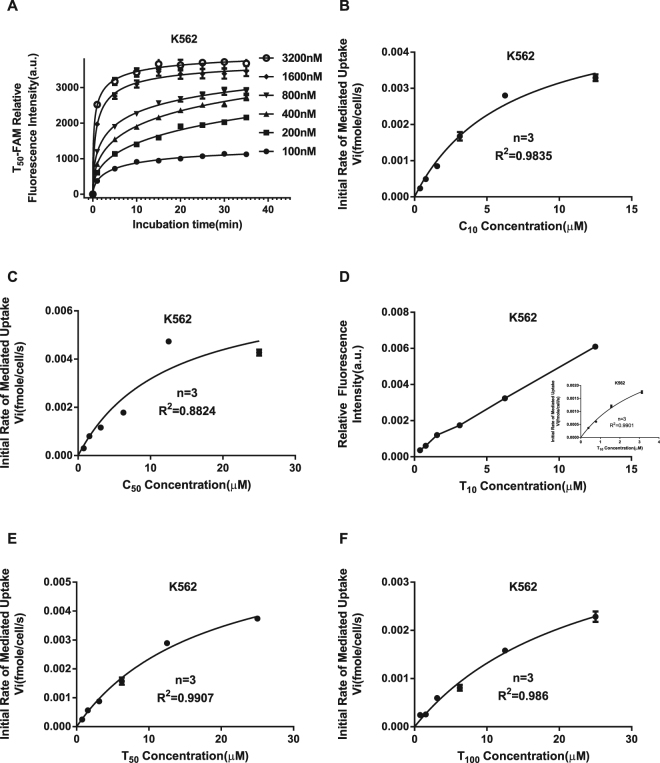
Kinetic analysis of the transmembrane transport of ODNs. Time courses of the transmembrane transport of various concentrations of T_50_-FAM were studied in K562 cells. As shown in Fig. 3A, the fluorescence intensity of the T_50_-FAM in K562 cells increased rapidly within 1 minute, exhibiting a linear increase. After 5 minutes, the increment of fluorescence slowed down significantly and gradually reached a plateau, indicating that T_50_ uptake had reached equilibration. Thus, we decided to estimate the initial rate of ODN transport at 30 s. The kinetic analysis of ODN (C_10_, C_50_, T_10_, T_50_, T_100_) transport, which was determined at different concentrations in K562 cells, is shown in Fig. 3(B–F). It is noteworthy that the uptake of T_10_ (Fig. 3D) depended on its concentration in the buffer. At a low concentration (insert graph), the uptake of T_10_ conformed to the Michaelis-Menten equation. However, at a high concentration, the uptake of T_10_ was similar to the pinocytosis of polyvinylpyrrolidone. Km and Vmax values are listed in Table 1. The experiments were performed in triplicate.

As shown in Fig. 3A, the fluorescence intensity of the various concentrations of T_50_-FAM-treated cells increased linearly and rapidly, within 1 minute. After 5 minutes, the increment of fluorescence slowed down significantly and gradually reached a plateau, indicating that the uptake of T_50_ had reached equilibrium. Therefore, we decided to estimate the initial rate of ODN transport at 30 s. The kinetics of ODN (C_10_, C_50_, T_10_, T_50_, T_100_) transport, which were determined in K562 cells at different concentrations of ODNs, are shown in Fig. 3B–F, and specific Km and Vmax values are listed in Table 1. It is noteworthy that the rate of T_10_ (Fig. 3D) uptake depended on its concentration in the buffer. At a low concentration (insert graph), T_10_ uptake conformed to the Michaelis-Menten equation; however, at a high concentration, it was similar to the pinocytosis of polyvinylpyrrolidone^23^.

**Table 1:** Estimates of Kinetic Parameters of ODN Uptake by K562 cells.

ODNs	Km ± SE (μM)	Vmax ± SE (amole/cell/s)	Vmax/Km (amole/cell/s/μM)
T_100_	24.12 ± 3.564	4.499 ± 0.396	0.19
T_50_	18.07 ± 1.946	6.570 ± 0.384	0.36
T_10_	3.78 ± 0.537	3.894 ± 0.35	1.03
C_50_	12.33 ± 4.374	7.093 ± 1.2	0.58
C_10_	6.512 ± 0.827	5.197 ± 0.32	0.80

Km and Vmax values were determined using the Michaelis-Menten equation from data presented in Fig. 3.

### Cellular localization of ODNs

**Figure 4:**
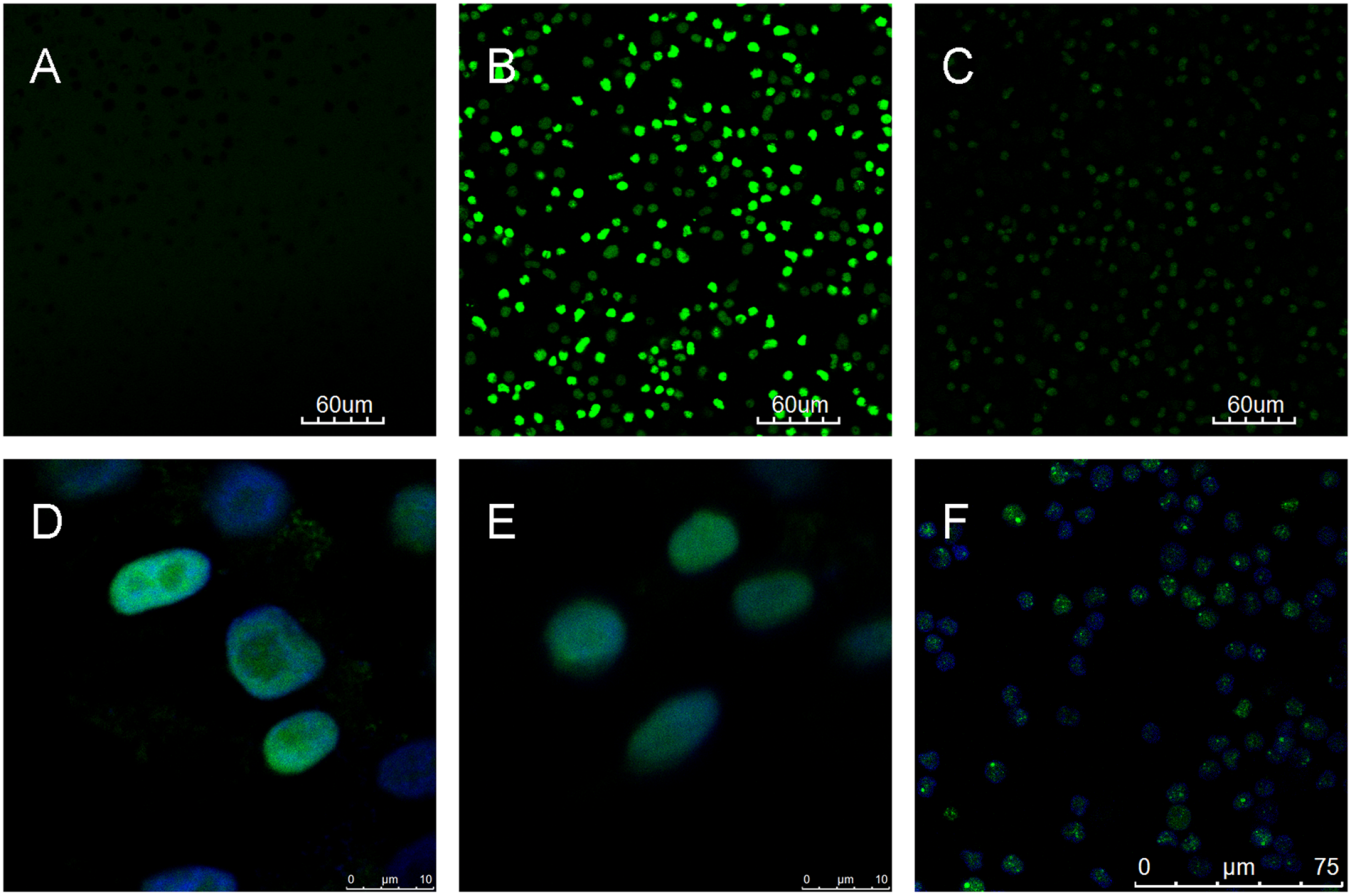
Confocal microscopy analysis of the cellular localization of various ODNs in the treated cells. (**A**) Control image with no fluorescence. (**B**) T_50_-FAM-treated cells, in which strong green fluorescence was detected on the cell membrane and in the cytoplasm. (**C**) C_50_-FAM-treated cells, in which moderate green fluorescence was detected in the cytoplasm. (**D**) G_10_-FAM-treated cells, in which strong green fluorescence co-localized with DAPI staining of the nucleus (blue fluorescence). (**E**) T_10_-FAM-treated cells, in which strong green fluorescence co-localized with DAPI staining of the nucleus (blue fluorescence). (**F**) TD08-FAM-treated cells, in which green fluorescence co-localized with DAPI staining of the nucleus (blue fluorescence). **A**,**B**,**C** and **F** were taken at 20X magnification; **D** and **E** were taken at 1000X magnification to show the nucleus. Images of FAM-labelled A_50_, C_10_, A_10_, A_1_, C_1_, T_1_, and G_1_-treated cells are not shown because of the very weak fluorescence intensity.

To be qualified for gene therapy applications, a delivery method should locate the target DNA in the cytoplasm or the nucleus, where the exogenous genes retain their functions^24^. However, in previously reported ODN cellular internalization processes, the exogenous DNA was targeted to the endocytic pathway and degraded in the lysosome^1^. To assess the therapeutic potential of ODN transmembrane transport, we determined the cellular localization of the ODNs in mixed clinical leukaemia cells (AML, B-ALL, and T-ALL) using confocal microscopy. In T_50_-FAM-treated cells, strong fluorescent signals were observed on the cell membrane and in the cytoplasm (Fig. 4B); in C_50_-FAM-treated cells, fluorescent signals were detected in the cytoplasm (Fig. 4C). However, in G_10_-FAM (Fig. 4D) and T_10_-FAM (Fig. 4E)-treated cells, the fluorescent signals were observed in the nucleus (stained with DAPI), indicating a nuclear localization of these two ODNs. In addition, aptamer TD08-FAM was also localized in the nucleus, as demonstrated by the overlapping green fluorescence and DAPI signals (Fig. 4F). The differential cellular localization of the various ODNs may be dependent on the length and specific sequence of an ODN. The precise underlying mechanism requires further investigation.

### Inhibition of ODN transport by dipyridamole but not by 4-nitrobenzylthioinosine (NBMPR)

**Figure 5:**
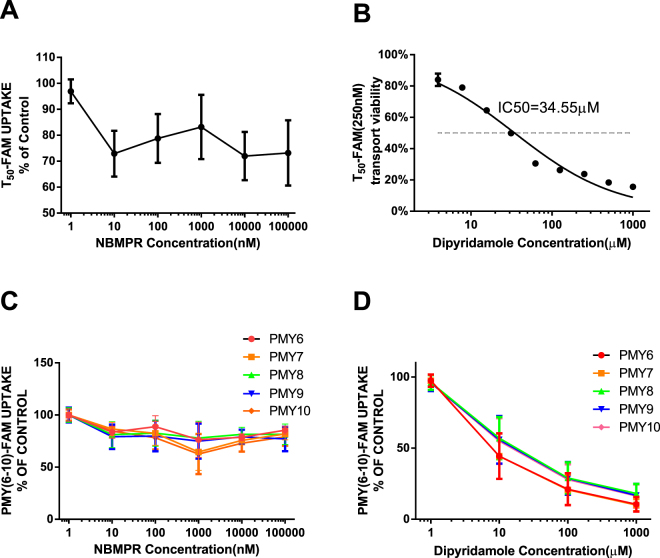
Inhibition of ODN transmembrane transport by dipyridamole but not by NBMPR. T_50_-FAM or PMY_6-10_-FAM uptake by cells could be blocked by pretreatment with dipyridamole (Fig. 5B,D) in a concentration-dependent manner (*p* < 0.05), but not by NBMPR (*p* > 0.05) (Fig. 5A,C). The uptake of T_50_ or PMY_6-10_ was largely inhibited by dipyridamole at a high concentration (1 mmol/L). When the concentration of T_50_-FAM was 250 nmol/L, the IC50 of dipyridamole was 34.55 μM. The experiment was repeated 3 times with similar results.

Because ENTs have been reported to transport nucleosides into cells and NBMPR or coronary vasodilator dipyridamole can inhibit the transport function^25,26^, we speculated that transmembrane transport of ODNs might be blocked by ENT inhibitor. To test this hypothesis, we pre-incubated clinical leukaemia cells (AML [n = 1], B-ALL [n = 1], and B-CLL [n = 1]) with NBMPR before addition of T_50_-FAM and PMY_6-10_-FAM. NBMPR clearly did not impede ODN transport (Fig. 5A and C). We also incubated cells with FAM-labelled T_50_ or PMY_6-10_ before or after treatment with the other ENT inhibitor, dipyridamole. Flow cytometry analysis showed that pretreatment of the cells with dipyridamole decreased the fluorescence signal of FAM-labelled T_50_ (Fig. 5B) or PMY_6-10_ (Fig. 5D), which suggested that T_50_ and PMY_6-10_ uptake was inhibited by dipyridamole in concentration-dependent manner (*p* < 0.05). When the concentration of T_50_-FAM was 250 nmol/L, the IC50 of dipyridamole was 34.55 µM.

Based on these experiments, we conclude that ODN transmembrane transport can be entirely inhibited by a high concentration of dipyridamole and therefore may differ from ENT-dependent nucleoside transport.

### Comparison of the efficiency of 3 kinds of transport methods and the transport of ssDNA (T_50_) and dsDNA (T = A_50_)

**Figure 6:**
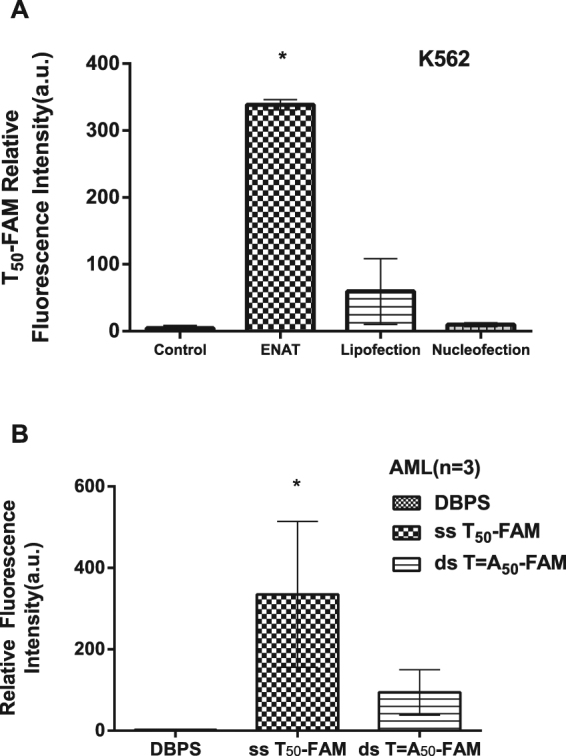
Comparison of the efficiency of 3 transport methods and the transport of ssDNA (T_50_) and dsDNA (T = A_50_). (**A**) The efficiency of FAM-labelled T_50_ delivery in K562 cells, as indicated by the fluorescence intensity, was significantly higher in the presence of ENAT (equilibrative nucleic acid transporter) compared with Lipofection or Nucleofection (*p* < 0.05). (**B**) The fluorescence intensity of clinical AML cells treated with FAM-labelled single-stranded T_50_ and double-stranded T = A_50_ vs control (incubated with DPBS). While strong fluorescent signals were detected in T_50_-treated cells, the signal intensity in T = A_50_ treated cells was very low (*p* < 0.05), indicating minimal dsDNA transmembrane transport. The experiments were performed in triplicate.

To further assess the therapeutic potential of ODN transmembrane transport in the clinical environment or research field, we compared the delivery efficiency between ODN transmembrane transport and transfection using Lipofection or Nucleofection in the K562 cell line. A leukaemia cell line was chosen for this experiment because it is easier to manipulate for transfection than clinical leukaemia cells. Equal numbers of cells were incubated with 250 nmol/L FAM-labelled T_50_ or transfected with the same amount of FAM-labelled T_50_ using Lipofection or Nucleofection. The delivery efficiency was quantified by flow cytometry. As shown in Fig. 6A, T_50_-FAM transport by vector-independent transmembrane transport showed a higher fluorescence intensity than those transfected by Lipofection or Nucleofection (*p* < 0.05). This result suggests that ODN transmembrane transport achieves a higher delivery efficiency compared with Lipofection or Nucleofection.

To examine whether double-stranded DNA (dsDNA) is also transported via vector-independent transmembrane transport, we tested FAM-labelled T = A_50_ in clinical AML cells. As shown in Fig. 6B, the transport efficiency of T = A_50_ was substantially decreased compared with single-stranded T_50_ (*p* < 0.05)_,_ which indicated that vector-independent transport of dsDNA was minimal. Compared with the ODN-treated cells, all control cells displayed minimal fluorescence intensity (*p* < 0.05).

### Signal transduction pathway involved in ODN transmembrane transport

**Figure 7:**
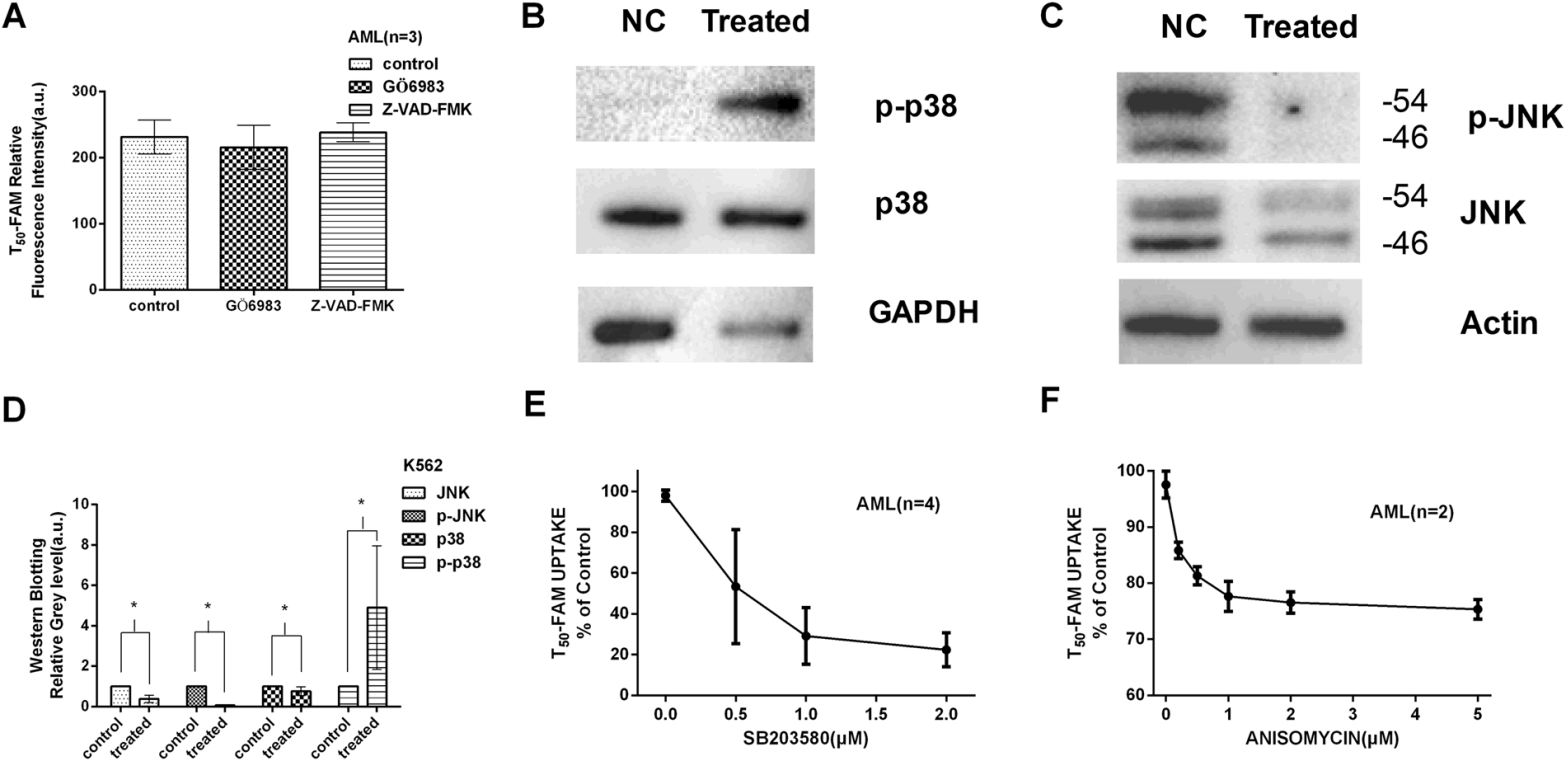
Signalling pathway involved in ODN transport. (**A**) The PKC inhibitor Gö6983 and apoptosis inhibitor Z-VAD-FMK had no effect on T_50_ transport (*p* > 0.05). (**B**) Cropped western blot images showing the total p38, p-p38 and GAPDH protein abundance, among which p-p38 was elevated and p38 was reduced in the treated sample compared with the NC (normal control) (both *p* < 0.05). (**C**) Cropped western blot showing total JNK, p-JNK and Actin protein abundance, among which both p-JNK and JNK were lower in the treated sample than the NC (*p* < 0.05), and p-JNK was much lower in treated sample than in the NC. Full-length blots and multiple exposures are provided in Supplementary file online. Quantification of the western blot bands in 7B and 7 C normalized to the internal and untreated controls are shown in (**D**), in which “*” represents a significant difference compared with the control at *p* < 0.05. In the blots, the samples are derived from the same experiment, and the gels/blots were processed in parallel. (**E**) Using the p38MAPK inhibitor SB203580, T_50_ uptake by the cells was largely decreased (*p* < 0.05). (**F**) Anisomycin, a potent activator of SPAKs/JNKs, should increase but slightly decreased the uptake of T_50_ (*p* > 0.05). These results suggested that rather than JNK, PKC or apoptosis signalling pathways, p38MAPK is involved in ODN transport. The experiments were performed in triplicate.

To further explore the signal transduction mechanism of ODN transport in cells, we investigated the influence on ODNs transport of several pathways inhibitors, such as the PKC inhibitor Gö6983 and the apoptosis inhibitor Z-VAD-FMK, which had no impact on T_50_ transport (*p* > 0.05) as shown in Fig. 7A. We also detected the phosphorylation of JNK and p38MAPK by western blotting (cropped blots were showed here and full-length and multiple exposures data are included in Supplementary file) and found that p38MAPK was lower but p-p38MAPK expression was clearly higher than the control group (Fig. 7B, *p* < 0.05), and the expression levels of both JNK & p-JNK were lower than in the control group (Fig. 7C, *p* < 0.05). Using the p38MAPK inhibitor SB203580, T_50_ uptake ability of the cells was largely decreased (Fig. 7E, *p* < 0.05). However, anisomycin, a potent activator of SPAKs/JNKs, did not increase the uptake of T_50_ (Fig. 7F, *p* > 0.05). Quantification values of the western blot bands normalized to internal and untreated control are shown in Fig. 7D. These results suggested that the p38MAPK rather than the JNK, PKC or apoptosis signalling pathway was involved in ODN transport.

## Discussion

It is well known that nucleoside transporters are classified into two categories, Na^+^-independent ENTs and Na^+^-dependent CNTs^27^. ENTs are a family of transmembrane proteins that comprises four members: ENT1-4. They mediate the bidirectional, concentration gradient-driven transport of nucleosides and their analogues into the cytoplasm. CNTs are also transmembrane proteins and have three members, CNT1-3^28^, which transport nucleosides and their analogues across the membrane in a unidirectional manner. Among all ENT members, ENT1 and ENT2 are sensitive to certain cardiovascular drugs, such as NBMPR, dipyridamole, and delazep^28,29^. They are also widely distributed in most tissue and cell types and have the ability to transport a broad range of purine and pyrimidine nucleosides into the cells^30,31^. In the present study, we showed that leukaemia cells could transport ODNs into the cytoplasm or nucleus in a sodium-independent (Fig. 1A) and energy-independent manner (Fig. 1C). Given that the activity of CNTs is cation-dependent and an active transport process^28^, we reasoned that CNTs were not involved in ODN transmembrane transport. The ODN transmembrane transport is insensitive to NBMPR, whereas it shows only weak sensitivity to dipyridamole with an IC50 of 35.44 µM for T_50_ (250 nmol/L, Fig. 5B), which is approximately 10–1000-fold higher than that of ENT1 or ENT2 for the relevant substrate^32,33^. In addition, the Vmax of ODN transport (Table 1) was 6 orders of magnitudes lower than the Vmax of substrate uptake for ENT1 and ENT2^33,34^; therefore, it is distinct from ENT1/2. Furthermore, ODN transport was cell membrane protein-dependent (Fig. 1B), pH-independent (Fig. 1D) and broadly permissive to substrates for ODNs. In contrast, ENT3 is primarily located in intracellular membranes such as the membranes of lysosomes and mitochondria^30,31^. Additionally, ENT4 is pH-dependent and specific for adenosine^35^, and hence is distinguished from ENT3 and ENT4. Moreover, some intracellular membrane proteins such as ANT (adenine nucleotide translocase, SLC25A4) and Sidt2, among others, which are located in mitochondrial membrane or lysosomal organelles and involved in ADP/ATP energy metabolism or RNA uptake^36,37^, also differ in protein location and functional aspects of ODN transport across the plasma membrane. The IC50 values of known proteins inhibited by dipyridamole, such as p-glycoprotein (IC50 1.5 ± 1.5 μM)^38^, breast cancer resistance protein (BCRP) (IC50 6.4 ± 0.9 μM)^39^, and ATP binding cassette transporter 5 (ABCC5) (Ki 1.2 μM or 5.5 μM)^40^, are one order of magnitude lower than that of ODN transport (IC50 35.44 µM). Furthermore, in contrast to these active transporter proteins, ODN transport is energy-independent (Fig. 1C).

We also compared the transport efficiency of ODNs composed of a single type of oligonucleotide (A, T, C, or G) with varying lengths and in different types of leukaemia cells. We found that ODNs with different compositions differed in their transport efficiency. Overall, among the 10-nucleotide ODNs, T_10_ exhibited the highest transport efficiency, whereas A_10_ had the lowest efficiency. In contrast, T chain ODNs with a length of 50 or 100 nucleotides demonstrated a higher transport efficiency than A chain and C chains with the same length (G chain ODNs were not investigated because of technical limitations). While this trend was consistent across the 4 types of leukaemia cells tested in this study, the transport efficiency of each individual ODN may differ in different cell types. The transport efficiency of different ODNs decreased in the following order for AML: T_100_ > T_50_ > T_10_ > C_50_ > G_10_ > C_10_ > A_10_ > A_50;_ for B-ALL: T_100_ > T_50_ > T_10_ > C_50_ > G_10_ > C_10_ > A_50_ > A_10;_ B-CLL: T_100_ > T_50_ > C_50_ > G_10_ > T_10_ > C_10_ > A_10_ > A_50;_ and for T-ALL: T_100_ > T_50_ > T_10_ > C_50_ > G_10_ > C_10_ > A_10_ > A_50._ We found that ODNs with different compositions showed the lowest fluorescence in T-ALL among all the tested clinical leukaemia cells, which is not consistent with the high mRNA expression of ENT1/2 in T-ALL^41^. This finding also confirmed that the high level of ENT1/2 mRNA expression was not correlated with the ODN transport activity in T-ALL. Additionally, the order of nucleotide preference of ODN transport differed from that of ENT1 and ENT2. Ward *et al*. has reported that the binding affinity of transporters for different nucleosides decreases in the following order for ENT1: adenosine > guanosine > isodine > uridine > thymidine >cytidine; and in the following order for ENT2: isodine > adenosine > uridine > thymidine > guanosine > cytidine^33^. Another study also reported that thymidine uptake is equally distributed between ENT1 and ENT2^42^. One possible explanation for the discrepancy in nucleotide preference between ODN transport and nucleoside transport is that cells possess two distinctive transport functions under different conditions; one is to transport nucleosides, and the other is to transport ODNs. Together, these data support that ODN transport is mediated by a novel nucleic acid transport system that is distinct from ENTs and CNTs. The protein identity of ODN transport is presently not known. Despite the lack of protein identity and literature evidence, we have named the ODN transporter equilibrative nucleic acid transporter (ENAT).

In the present study, we were particularly concerned about the transmembrane transport of A_50_ because of possible interference from intracellular mRNA poly(A) tails. To eliminate such interference, we pre-treated the cells with T_50_ before the incubation with A_50_. Our results demonstrated that the cells were able to transport A_50_ into the cytoplasm, and pre-incubation with T_50_ increased the transport efficiency. Taken together, ENAT was able to transport ODNs with varying sequences, although some sequence-dependent differences in transport efficiency were observed. Furthermore, we confirmed that ENAT was able to transport random sequence ssDNA and aptamers into the cytoplasm or the nucleus. Overall, ENAT is broadly selective for pyrimidine and purine nucleotide strands and is a highly efficient transport system distinct from endocytosis, leading to nucleic acid degradation in the lysosome^1^. These findings suggest potential research or therapeutic applications for ENAT, particularly in the gene therapy field. Currently, gene therapy primarily relies on viral vectors to deliver various DNA sequences. However, viral vectors have potential side effects such as virus-mediated pathogenicity, oncogenicity, or immunogenicity, as well as the need to reserve longer DNA fragments to accommodate the viral genes. Therefore, ENAT would be very beneficial for clinical usage because it can deliver ODNs into the cytoplasm or the nucleus in the absence of vectors. Furthermore, ENAT demonstrated a higher delivery efficiency than transfection using Lipofection or Nucleofection. However, although it may not be suitable for the transport of double-stranded ODNs (Fig. 6B), ENAT provides a potentially safe and effective approach for ODN transport in gene therapy.

It is also important to understand the regulatory mechanism of ENAT to determine its potential clinical value. Recently, two studies indicated that ENT inhibition prevented p38MAPK and c-JNK activity and EC barrier dysfunction^43,44^. Huang M *et al*. found that nucleoside uptake of K562 cells was prevented by the inactive p38MAPK inhibitor SB202474, which suggested that nucleoside uptake inhibition by SB203580 was independent of p38MAPK inhibition but linked to the compound itself^45^. A study suggested that the phosphorylation of multiple pathways, such as PKC and PKA, was involved in ENT1^46^. We speculate that the pathways responsible for modulating ENTs are related to ENAT. Thus, we investigated the influence of different signalling pathway inhibitors (SB203580 for p38MAPK, Gö6983 for PKC and Z-VAD-FMK for apoptosis) on ENAT. The results showed that the inhibitor of p38MAPK weakened the activity of ENAT, but inhibition of PKC and apoptosis had no effect (Fig. 7F). Western blotting results showing increased p-p38MAPK and decreased p38MAPK but decreased p-JNK and JNK, compared with the control sample, also support the involvement of p38MAPK phosphorylation in ENAT. These results facilitate our understanding of the mechanism by which ENAT is modulated under certain condition, i.e., solution A treatment (recipe pending patent application).

In conclusion, we demonstrated ODN transmembrane transport occurs via ENAT in clinical leukaemia cells; however, the exact underlying mechanisms require further investigation. Other areas of future research include determination of the transport function regulation of ENAT and whether oligoribonucleotides can be transported across the membrane in a vector-independent manner.

## Materials and Methods

### Synthesis of ODNs and random sequence ODNs from library amplification

The random sequence ssDNA library, PCR primers, fluorescein (FAM)-labelled aptamers (sgc 6, sga 16, TD 08), ODNs composed of a single type of nucleotides with varying lengths (A_1_, T_1_, C_1_, G_1_, A_10_, T_10_, C_10_, G_10_, A_50_, C_50_, T_50_, T_100_) labelled with 5′-FAM and ODNs (C_10_, C_50_, T_10_, T_50_, T_100_) labelled with 5′-FAM and phosphorothioate at 3 base of both ends were synthesized by Shanghai Biological Engineering Co., Ltd. (Shanghai, China). Phosphorothioate A_10_ and A_50_ were not synthesized because A_10_ and A_50_-FAM exhibited very dim fluorescence in cells. G_50_ and phosphorothioate G_10_ were not synthesized due to technical limitations.

The random sequence ssDNA library contained the following sequences: 5′-GCAATGGTACGGTACTTCC (50 N) CAAAAGTGCACGCTACTTTGCTAA-3′, where “50 N” represented random sequences of 50 nucleotides. The ssDNA library was amplified by PCR using the following forward primer 5′-GCA ATG GTA CGG TAC TTC C-3′ and reverse primer 5′-TTA GCA AAG TAG CGT GCA CTT TTG-3′. Hot Master Taq DNA polymerase, 10 × PCR buffer, and dNTPs were purchased from TIANGEN BIOTECH Co., Ltd. (Beijing, China). The PCR products were purified by agarose gel electrophoresis and sequenced by Beijing Nohezhiyuan Co., Ltd. (Beijing, China). The 5 sequences with the highest abundance (PMY_6-10_) were further synthesized for FAM labelling in large quantities.

### Cell lines, culture conditions, and transfection

The human chronic myeloid leukaemia cell line K562 was obtained from the Cell Research Center, Centre South University XiangYa School of Medicine. K562 cells were cultured in RPMI-1640 medium (GIBCO BRL, Burlington, MA, USA) containing 10% foetal bovine serum at 37 °C in humidified air supplied with 5% CO_2_.Cells in the logarithmic growth phase were harvested and seeded in 6-well plates at a concentration of 1 × 10^6^/ml. Cells were transfected with T_50_-FAM (final concentration 250 nmol/L) using Lipofection or Nucleofection. Lipofection was performed using the RiboFECT™ CP Transfection Kit (Guangzhou RiboBio Co., Ltd, Guangzhou, China) according to the manufacturer’s instructions. Nucleofection was performed using the Amaxa™ Human Stem Cell Nucleofector™ kit 2 (Amaxa Biosystems, Gaithersburg, MD, USA) according to the manufacturer’s instructions. Cells were collected 24 hours after Lipofection and immediately after Nucleofection for flow cytometry (FACSCalibur or FACSCanto II, BD Bioscience, San Jose, CA, USA) to determine the transfection efficiency.

### Cell isolation and treatment

Human clinical leukaemia cells were isolated from bone marrow or peripheral blood cell samples from 28 patients who had signed the informed consent. The patients ranged in age from 1 to 76 years, and 14 of them were male. Of the 28 patients, 16 had acute myeloid leukaemia (AML), 5 had B-cell acute lymphoblastic leukaemia (B-ALL), 4 had B-cell chronic lymphocytic leukaemia (B-CLL), and 3 had T-cell acute lymphoblastic leukaemia (T-ALL). The study protocol was approved by the institutional review boards at Xiangya Hospital, Central South University, and the research design and methods were performed in accordance with the regulatory requirements and procedures regarding human subject protection laws such as GCP and ICH-GCP. Mononuclear cells were separated using lymphocyte separation medium (Sigma-Aldrich, St Louis, MO, USA) following routine procedures. In brief, clinical samples were gently added to equal volumes of lymphocyte separation medium in centrifuge tubes and centrifuged at 1500 revolutions per minute (rpm) for 5 minutes. The middle layer containing the white blood cells (between the plasma layer on the top and the separation medium at the bottom) were collected by aspiration, washed once, and re-suspended in Dulbecco’s phosphate-buffered saline (DPBS; Shanghai Lifei biological technology Co., Ltd, Shanghai, China).

Clinical cells and K562 cells were divided into treatment and control groups. Treatment cells were incubated for 10 minutes in solution A (recipe pending patent application) at a 1:9 ratio and then washed 3 times and re-suspended in DPBS. Control group cells were also washed 3 times and suspended in DPBS. Both the treatment and control groups were adjusted to a concentration of 1 × 10^6^/ml.

Two buffers (sodium and sodium-free solution), which have been described previously, i.e., 1 mM MgCl_2_, 5 mM KCl, 10 mM HEPES, 25 mM CaCl_2_, 137 mM choline chloride or NaCl^22^, were prepared for ODN transport to evaluate sodium dependence. Treatment group cells were washed 3 times and re-suspended in the two buffers described above. All the ODNs were also prepared in two buffers at a concentration of 500 nmol/L. The same volume of cells and ODNs were mixed in the dark at 4 °C for 30 minutes for flow cytometry analysis. As shown in Fig. 1A, ODN transport was sodium-independent, and thus, all subsequent experiments were conducted in DPBS or sodium buffer.

To determine whether ODN transport was membrane protein-dependent, trypsin was added to the treated cells at a concentration of 0.25%. After 2−5 minutes of incubation, trypsin was inactivated by addition of foetal bovine serum, and then the cells were washed 3 times and re-suspended in DPBS. Finally, the cells were incubated with T_50_-FAM in the dark at 4 °C for 30 minutes for flow cytometry analysis.

To examine whether ODN transport was energy dependent, a portion of the treatment group cells was pre-incubated at 4 °C with 10 mM sodium azide and 6 mM 2-deoxy-D-glucose for 1 hr to delete cellular ATP^47^. The other cells served as a control. The two cell groups were then incubated with T_50_-FAM in the dark at 4 °C for 30 minutes for flow cytometry analysis.

To investigate whether ODN transport was pH-dependent, the treatment group cells were washed 3 times and re-suspended in pH 5 to pH 8 phosphate buffer. The cells were then incubated with T_50_-biotin in the dark at 4 °C for 20 minutes. The cells were washed once with pH 5–8 phosphate buffer and incubated with streptavidin-PE (BD Bioscience, Bedford, MA, USA) in the dark at 4 °C for 20 minutes. After another wash, the cells were re-suspended in pH 5–8 phosphate buffer for flow cytometry analysis.

### ODN Transmembrane transport

Various ODNs or dsDNA labelled with FAM at a concentration of 500 nmol/L were added to the treated and control cells, and both groups were incubated in the dark at 4 °C for 30 minutes. After incubation, the cells were washed and re-suspended in DPBS for flow cytometry or confocal microscopy analysis. For confocal microscopy, pre-DAPI staining (100 ng/ml for 20 minutes) was performed followed by re-suspension in DPBS. A FluoView^TM^ FV1000 laser confocal microscope (Olympus, Tokyo, Japan) was used.

To determine the linearity of ODN transport, the same volume of cells and various concentrations of T_50_ -FAM (0.1–3.2 μmol/L) were mixed in the dark at 4 °C, and then, T_50_ transport into cells was examined at different time points up to 30 minutes by flow cytometry analysis.

Transport kinetic assays were performed as described previously^48^. Flow cytometry was used to analyse the uptake of phosphorothioate ODNs, in which the oxygen atom of the phosphate group was replaced with a sulphur atom to resist nuclease degradation^49^. Briefly, the cells were suspended in PBS, and then, the same volume of cells and various concentrations of phosphorothioate ODNs were mixed and immediately analysed by flow cytometry. The relative fluorescence intensity of the cells at 30 s was converted into MEF by comparing the single cell relative units of the channel number with the fluorescence of SPHERO^TM^ Easy Calibration Fluorescent Particles (Catalogue No. ECFP-FI-5K, Spherotech, Lake Forest, IL, USA) under the same conditions. Treated cells (total uptake) diminish the control cells (non-specific binding) are defined as mediated uptake.

### Pre-incubation with dipyridamole and NBMPR

Treated cells were pre-incubated with various concentrations (from 1 μmol/L to 1 mmol/L) of dipyridamole (Dalian Mellon biological technology Co. Ltd, Dalian, China) and (from 1 nmol/L to 100 μmol/L) NBMPR (Sigma-Aldrich, St Louis, MO, USA), a hENT1 inhibitor, for 30 minutes before incubation with T_50_-FAM or PMY_6-10_-FAM for 30 minutes. The cells were then re-suspended for analysis by flow cytometry or confocal microscopy.

### Protein extraction and Western Blotting

First, K562 cells were incubated for 10 minutes in solution A, which contained 1 × protease inhibitor and 1 × PhosStop cocktail (Roche, France), and then the treated cells were lysed in RIPA buffer (Thermo Fisher, Waltham, MA, USA). Control cells were lysed in RIPA buffer in the presence of 1 × protease inhibitor and 1 × PhosStop cocktail for 20 minutes. After sonication three times, the lysed cell product was centrifuged at 12000 rpm for 10 minutes at 4 °C. The protein concentration was measured using a Pierce® BCA protein assay kit (Thermo Fisher, Waltham, MA, USA). Western blotting analysis was performed as described previously^50^. Anti-human actin, GAPDH, p38MAPK, p-p38MAPK, JNK, and p-JNK were obtained from Santa Cruz (Santa Cruz, CA, USA).

### Signalling pathway inhibition or activation

Before incubation with T_50_-FAM for 30 minutes, the treated cells were pre-incubated with the p38MAPK inhibitor SB203580 (0.5,1, 2 µmol/L) for 1 hr; a potent activator of SPAKs/JNKs, anisomycin (0.2, 0.5, 1, 2, 5 µmol/L) for 3 hr; the PKC inhibitor Gö6983 (100 nmol/L) for 1 hr; or the apoptosis inhibitor Z-VAD-FMK (50 nmol/L) for 1 hr. The cells were finally re-suspended for analysis by flow cytometry. (SB203580, Anisomycin, Gö6983 and Z-VAD-FMK were purchased from MedChem Express, Monmouth Junction, NJ, USA.)

### Statistical analysis

All data are presented as the mean ± SD except where indicated. Paired or multiple group unpaired data were analysed with the Student’s paired t test or two-way ANOVA, respectively. Km, Vmax and IC50 values were calculated by fitting the data to a non-linear regression Michaelis-Menten equation or dose-response inhibition curve, respectively. All analyses, linear regression lines and curves were defined using GraphPad software 6.0 (GraphPad Software Incorporated, San Diego, CA, USA). *p < 0.05 was considered a statistically significant difference.

### Data availability

All data generated or analysed during this study are included in this published article (and its Supplementary Information files).

## Electronic supplementary material

Supplementary Information
