# Supplementary material for: Vector-independent transmembrane transport of oligodeoxyribonucleotides involves p38 mitogen activated protein kinase phosphorylation
Source: Sci Rep. 2017 Oct 19;7:13571. doi: 10.1038/s41598-017-14099-0 (PMC5648841; doi:10.1038/s41598-017-14099-0)
Supplement: Supplementary file 1 — Supplementary Information [file 41598_2017_14099_MOESM1_ESM.pdf]

Vector-independent transmembrane transport of oligodeoxyribonucleotides involves p38 mitogen activated protein kinase phosphorylation

- Minyuan Peng [1], Yanming Li [2], Jian Zhang [3], Yong Wu[1], Xiaoyang Yang [1], Ye Lei [4], Mao Ye [5], Jing Liu [6], Xu Han[6], Yijin Kuang[6], Xielan Zhao [1], Fangping Chen [1,*]

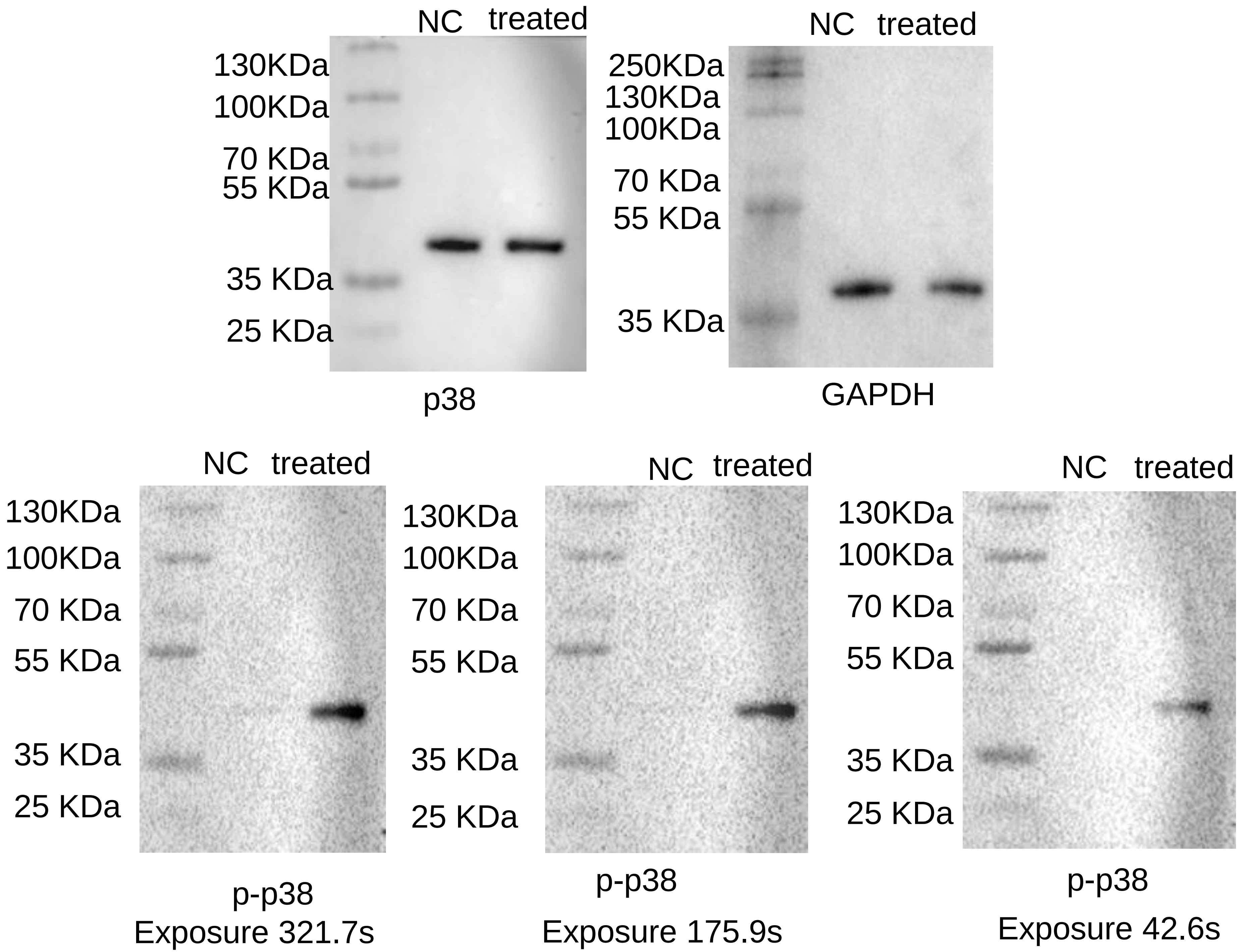
NC
treated
130KDa
100KDa
70 KDa
55 KDa
35 KDa
25 KDa
p38
NC
treated
250KDa
130KDa
100KDa
70 KDa
55 KDa
35 KDa
GAPDH
NC
treated
130KDa
100KDa
70 KDa
55 KDa
35 KDa
25 KDa
p-p38
Exposure 321.7s
NC
treated
130KDa
100KDa
70 KDa
55 KDa
35 KDa
25 KDa
p-p38
Exposure 175.9s
NC
treated
130KDa
100KDa
70 KDa
55 KDa
35 KDa
25 KDa
p-p38
Exposure 42.6s

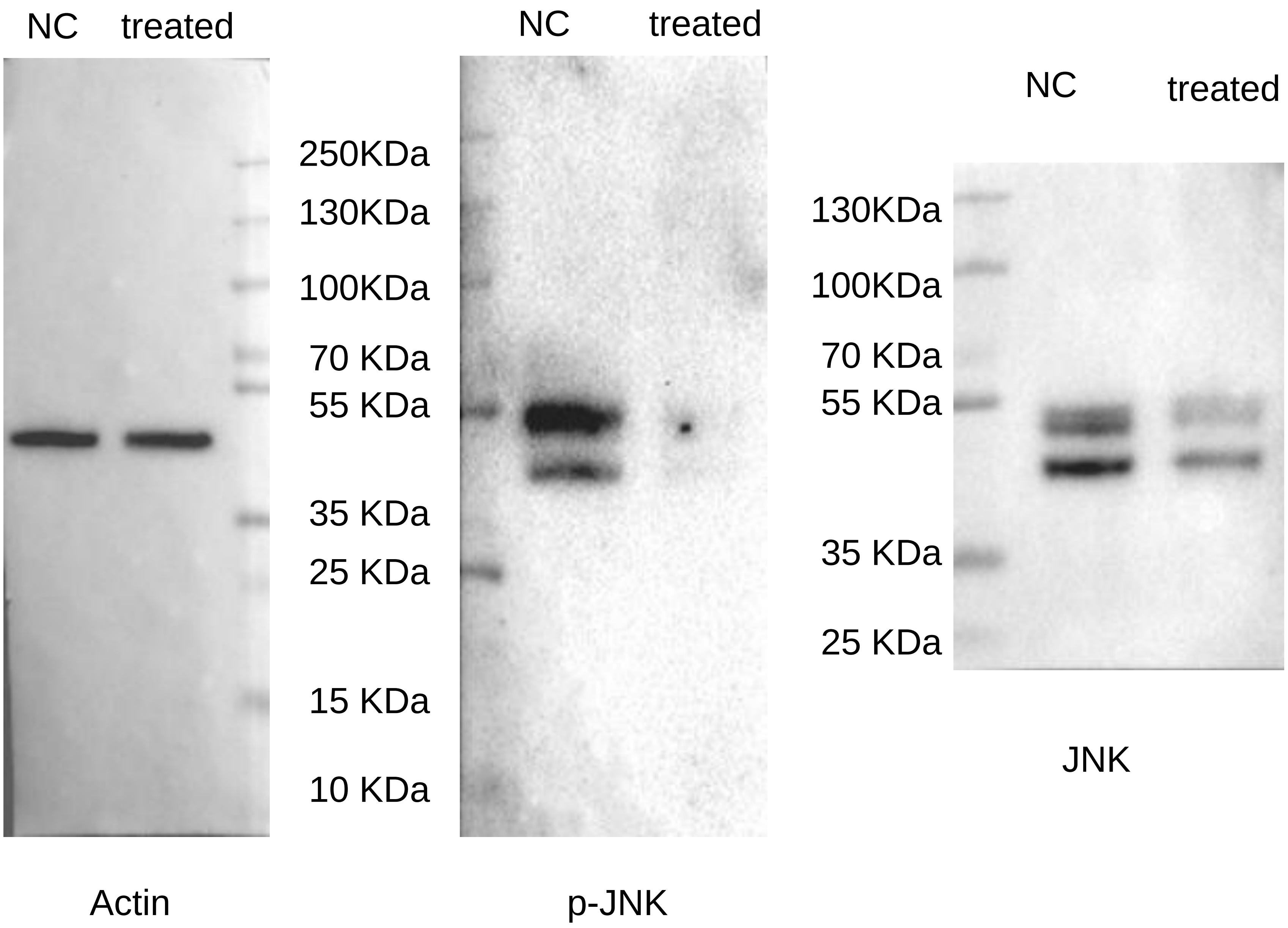
NC
treated
Actin
NC
treated
250KDa
130KDa
100KDa
70 KDa
55 KDa
35 KDa
25 KDa
15 KDa
10 KDa
p-JNK
NC
treated
130KDa
100KDa
70 KDa
55 KDa
35 KDa
25 KDa
JNK
